# Mathematical Modeling of the Effect of Water Splitting on Ion Transfer in the Depleted Diffusion Layer Near an Ion-Exchange Membrane

**DOI:** 10.3390/membranes10020022

**Published:** 2020-01-31

**Authors:** Victor Nikonenko, Mahamet Urtenov, Semyon Mareev, Gérald Pourcelly

**Affiliations:** 1 Department/school, Kuban State University, 149 Stavropolskaya St., 350040 Krasnodar, Russia; urtenovmax@mail.ru (M.U.); mareev-semyon@bk.ru (S.M.); 2 Institut Européen des Membranes, UMR 5635 (CNRS-ENSCM-UM), Université Montpellier, Place E. Bataillon, F-34095 Montpellier, France; gerald.pourcelly@umontpellier.fr

**Keywords:** ion exchange membranes, concentration polarization, overlimiting current, ion transfer, mathematical modeling

## Abstract

Water splitting (WS) and electroconvection (EC) are the main phenomena affecting ion transfer through ion-exchange membranes in intensive current regimes of electrodialysis. While EC enhances ion transport, WS, in most cases, is an undesirable effect reducing current efficiency and causing precipitation of sparingly soluble compounds. A mathematical description of the transfer of salt ions and H^+^ (OH^−^) ions generated in WS is presented. The model is based on the Nernst–Planck and Poisson equations; it takes into account deviation from local electroneutrality in the depleted diffusion boundary layer (DBL). The current transported by water ions is given as a parameter. Numerical and semi-analytical solutions are developed. The analytical solution is found by dividing the depleted DBL into three zones: the electroneutral region, the extended space charge region (SCR), and the quasi-equilibrium zone near the membrane surface. There is an excellent agreement between two solutions when calculating the concentration of all four ions, electric field, and potential drop across the depleted DBL. The treatment of experimental partial current–voltage curves shows that under the same current density, the surface space charge density at the anion-exchange membrane is lower than that at the cation-exchange membrane. This explains the negative effect of WS, which partially suppresses EC and reduces salt ion transfer. The restrictions of the analytical solution, namely, the local chemical equilibrium assumption, are discussed.

## 1. Introduction

Electrodialysis (ED) and electrodialysis reversal (EDR) are widely used in different applications including desalination of brackish waters, such as river waters and waters in agriculture [1,2,3,4,5,6], mine water disposal [7], food industry applications (dairy, wine, separation of amino acids, etc.) [8,9,10,11], and other applications. The electromembrane technique is often used together with other membrane methods, such as reverse osmosis and ultrafiltration [12,13]. It is clear that the use of intensive current regimes in ED operation reduces the area of ion exchange membranes (IEMs), hence, the investment costs of the process. Indeed, these regimes are used more and more often in such kinds of ED as shock electrodialysis [14,15], electrodeionization [16,17], and microfluidic devices [18,19,20]. Although with increasing current density, the energy costs increase, lower investment costs allow one to obtain an economic effect when applying overlimiting currents [21].

However, in the overlimiting current regime, there are current-induced phenomena not occurring at low current densities. The main phenomena which take place at *i ≥ i_lim_* are electroconvection [22,23,24,25,26] and water splitting [27,28,29,30]. Electroconvection is the fluid transfer occurring under the action of an electric force on the space charge in solution. The main mechanism of EC is electroosmotic slip, which takes place when an electric force is applied to the space charge in the depleted solution located at the membrane surface [31]. In the literature, two kinds of electroosmosis in membrane systems are distinguished: the electroosmosis of the first kind, when the space charge exists independently on the applied current, and electroosmosis of the second kind, when an extended SCR is formed by an (overlimiting) current [24,32]. EC occurring in the first case is also called equilibrium electroconvection [33], while that in the second case is called non-equilibrium electroconvection [34,35]. Electroconvection is the main effect enhancing mass transfer in membrane systems in intensive current regimes. The micrometer-scale electroconvective vortices mix the fluid near the membrane surface [36,37,38,39]. Often this effect of EC mixing, which leads to the formation of a flattened concentration profile in the depleted region adjacent to the membrane surface, is interpreted as the reduction in the effective thickness of the solution diffusion layer [22,38,40,41]. Interestingly, the dominant diffusion zone is offset from the membrane surface, so that the diffusion layer is no longer the boundary one.

Unlike electroconvection, which improves the ED performance, generation of H^+^ and OH^–^ ions, with the exception of some cases where the pH is adjusted to a targeted value [42], is an undesirable process during electrodialysis. This phenomenon results in a decrease in current efficiency and a change in the pH of the solutions. The latter often causes precipitation of sparingly soluble salts and their deposition on the membrane surface and sometimes within the membrane pores (membrane scaling). In addition, under pH changes, the deposition of organic matter is also possible, the phenomenon known as fouling [43]. However, it was shown in a number of papers [44,45,46] that EC not only enhances the mass transfer rate, but also decreases the water splitting rate, and, hence, reduces scaling and fouling.

As for the mechanism of water splitting, the studies of Simons [47,48], Timashev et al. [49], Mafé et al. [50], Strathmann et al. [51], Zabolotsky, Sheldeshov et al. [28,52], and other authors [53,54,55] established that most of the H^+^ and OH^−^ ions are generated in proton-transfer reactions between the membrane functional groups and water in a thin, a few nanometers thick, boundary layer of the membrane. The weaker the functional ionogenic groups, the easier the generation of H^+^ and OH^–^ ions. The strong electric field in the reaction layer can increase the overall effective water splitting rate constant by several orders [51] via facilitating the favorable water molecules orientation and accelerating the rate of evacuation of the hydrogen and hydroxyl ions from the reaction layer (the second Wien effect) [50]. The amount of the H^+^ and OH^–^ ions generated in the depleted boundary solution is essentially less, since the rate constant of water dissociation in free solution (equal to 2 × 10^−5^ s^−1^) is much lower than the effective rate constant in the membrane, *k*_d_^*^, where water splitting is facilitated by the proton-transfer reactions. For example, in the case of an anion-exchange membrane with tertiary amino groups, *k*_d_ *≈* 1 s^−1^ [28]. However, as it was shown recently by Urtenov et al. [56], the amount of the H^+^ and OH^−^ generated in the depleted solution can be significant at high voltages of the order of 10 V over a membrane. As it was found in [56], the water-splitting reaction takes place within overall SCR, which can reach up to 10 µm at high voltages.

There is another effect related to water splitting first considered by Kharkatz [57,58]. The water ions generated at the membrane surface affect the electric field in the depleted diffusion layer and, hence, the salt ion fluxes. Thus, the OH^−^ ions generated at a cation-exchange membrane (CEM) and moving into the depleted solution attract the salt cations from the bulk and increase their flux towards the membrane surface. This effect, called the exaltation of the limiting current [57], is described in the case of a 1:1 electrolyte and neutral bulk solution (pH = 7) by a simple relation [29,57]:

(1) $$I_{+}=\frac{2D_{+}C_{+}^{0}F}{\delta }+\frac{D_{+}}{D_{OH}}I_{w},$$

where $I_{+}$ is the partial current density of the salt counterion (assuming to be a cation at a CEM), $I_{w}$ and *D_OH_* are the current density and the diffusion coefficient of the water ion (OH^–^ in the case of the cation-exchange membrane) in the depleted diffusion layer; *δ* is the thickness of the diffusion layer. When obtaining Equation (1), it is assumed that the partial current density of the Cl^−^ ions through the membrane is negligible. Note that if $I_{w}=0$ (no water splitting occurs), Equation (1) reduces to the well-known Peers equation [29]: $I_{+}=\frac{2D_{+}C_{+}^{0}F}{\delta }.$

In the case of an anion-exchange membrane (AEM), the hydrogen ions move from the membrane interface into the solution bulk. Hence, *D_H_* should replace *D_OH_* in Equation (1). The membrane is supposed not permeable for salt co-ions. The first term represents the contribution of the electrodiffusion through the diffusion layer; the second term is the exaltation current, which is obviously zero in the absence of the water splitting.

The salt ion transport under the condition of the water splitting was also considered by Zholkovsky [59] and Gnusin [60] when applying the local electroneutrality. However, it is of great interest to study the salt and water transport by using the Poisson equation instead of the electroneutrality condition. There are only a few papers, those of Volgin and Davydov [61] and Femmer et al. [54,62] reporting the numerical solutions of the Nernst–Planck and Poisson (NPP) equations in conditions of water splitting. However, as far as we know, there have been no attempts to obtain an analytical solution to this problem in the case of monopolar membranes, although there are a few analytical methods developed for the solution of the NPP equations for ion transport in membrane systems in the case of the absence of water splitting [63,64,65,66,67,68].

In this paper, we propose an analytical solution of the NPP equations for describing the ion transport in the depleted diffusion layer adjacent to a monopolar ion-exchange membrane in conditions of water splitting at the solution/membrane interface. We apply the same mathematical method that was developed in [64] for solving the NPP equations in the depleted diffusion layer near an IEM in the case of a strong 1:1 electrolyte. The method consists of obtaining approximate solutions in different zones of the diffusion layer, followed by “stitching” the obtained solutions. The approximate solution is compared with the results of numerical computations.

A theoretical analysis of the effect of water splitting on salt transport in the depleted diffusion layer is carried out.

## 2. Mathematical Description 

### 2.1. Formulation of the Problem. Governing Equations and Additional Conditions

**Figure 1:**
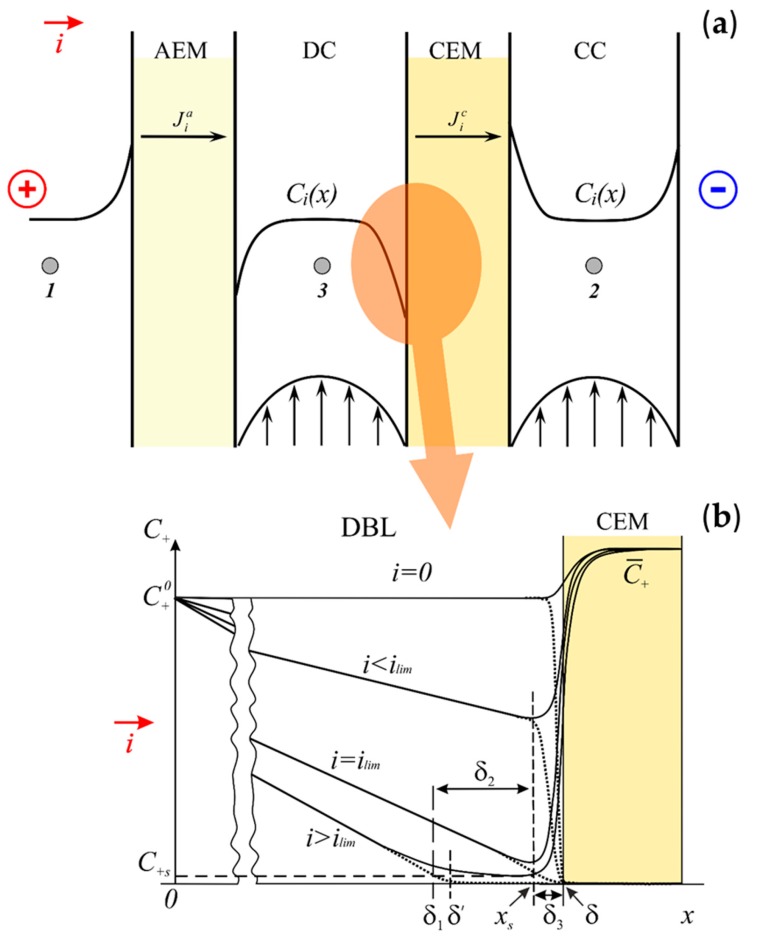
An electrodialysis cell with anion-exchange membranes (AEM) and cation-exchange membranes (CEM) forming desalination (DC) and concentration (CC) compartments (**a**); concentration profiles of a salt counterion in the diffusion boundary layer (DBL) at a CEM schematically shown at different current densities (**b**). “1”, “2”, and “3” indicate the points between which the potential drop is determined. *δ*′ is the effective DBL thickness; *δ*_1_, *δ*_2_, and *δ*_3_ are the thicknesses of the electroneutral zone, extended space charge region (SCR), and the equilibrium part of the electric double layer, respectively, of the DBL. Redrawn from [68].

Figure 1a shows an electrodialysis cell consisting of alternating AEM and CEM forming desalination (DC) and concentration (CC) compartments. An anode, bounding the membrane stack on the left, and a cathode, bounding the stack on the right, are used to supply an electric current through the membranes. When formulating a 1D model describing ion transport and water splitting in the system, we consider a fragment of the overall system comprising one ion-exchange membrane and two identical bathing solutions of 1:1 electrolyte with concentration $C_{1}^{0}$. It is assumed that in the considered cross-section, there are two diffusion layers at both sides of the membrane (a CEM is considered for definiteness). The thickness of the diffusion layers, *δ*, represents a model parameter; the determination of its value from the experimental I–V curves will be described below. Only the depleted diffusion layer adjacent to the left side of the membrane is considered in detail. Figure 1b shows schematically the concentration profiles of the salt counterion in this diffusion layer. 

Let a direct current of density *I* flow normally to the membrane surface. The fluxes *J_i_* of salt cations (+) and anions (−), as well as the fluxes of the H^+^ and OH^−^ ions in the diffusion layer, are described by the Nernst–Planck equation:

(2) $$J_{i}=-D_{i}\left(\frac{dC_{i}}{dX}-z_{i}C_{i}\frac{FE}{RT}\right),\;i=“+”,\;“-“,\;“H”,\;“\mathrm{OH}”,$$

where *D_i_*, *z_i_*, and *C_i_* are the diffusion coefficient, charge, and concentration of ion *I*, respectively; *E* is the electric field, the notations *F*, *R,* and *T* refer to the Faraday constant, gas constant, and absolute temperature, respectively. The normal coordinate *X* takes its zero value in the bulk solution (to the left of the membrane), *X* = *δ* on the membrane surface, Figure 1b. The case of 1:1 electrolyte is considered, hence z_+_ = 1, z_–_ = −1.

The space charge, which is due to unbalance of the total amounts of cations and anions in a volume element, is connected with the electric field by the Poisson equation:

(3) $$\varepsilon_{0}\varepsilon \frac{dE}{dX}=F\left(C_{+}-C_{-}+C_{H}-C_{OH}\right),$$

where $\varepsilon_{0}$ is the permittivity of the vacuum, ε is the relative dielectric permeability in solution assumed constant (ε = 78 at *T* = 298 K).

The current density *I* results from the ionic fluxes:

(4) $$I=F\left(J_{+}-J_{-}+J_{H}-J_{OH}\right),$$

In steady-state, the salt ion fluxes do not vary with the coordinate *X*. The fluxes of H^+^ and OH^−^ ions are variable due to the generation or recombination of these ions, however, the sum $J_{W}=J_{H}-J_{OH}$ remains constant because the number of H^+^ ions appearing or disappearing at a point *X* is equal to the corresponding number of OH^−^ ions.

The rate of the H^+^ and OH^−^ ions’ generation per unit volume of solution in the diffusion layer, $R_{H}$, can be written as follows:

(5) $$R_{H}=R_{OH}=k_{d}C_{w}-k_{r}C_{H}C_{OH},$$

where *k_r_* is the rate constant for the recombination reaction equal to 1.1 × 10^11^ L·mol^−1^·s^−1^, $C_{w}$ = 55.6 mol·L^−1^ is the water concentration. The rate constant for the dissociation reaction, $k_{d}$, is generally a function of the electric field, *E*. This dependence is often described by an exponential function [49,50,52]:

(6) $$k_{d}\left(E\right)=k_{d}\left(0\right)e^{\beta E},$$

where coefficient β should be of the order of 10^−8^ m·V^−1^ [52]. The estimations made by Strathmann [51] show that the electric field in the interface layer of the bipolar membrane is in the range 6 × 10^8^−9 × 10^8^ (V·m^−1^). Thus, the $k_{d}\left(E\right)/k_{d}\left(0\right)$ ratio in bipolar membranes can be equal to several tens of times or more. However, in the depleted solution adjacent to a monopolar membrane, this ratio is not much greater than 1, since the electric field at the interface is about two orders of magnitude lower, as we will see in Section 3. The reason is that in the bipolar membrane, the main potential drop occurs in the bipolar junction, while in the solution/monopolar membrane system, the region with the highest resistance is the extended SCR in the depleted solution. In addition, there is a relationship between the values of the electric field in the interface between solution (*E_m_*) and membrane (${\bar{E}}_{m}$), $\bar{\varepsilon }{\bar{E}}_{m}=\varepsilon E_{m}$ (Equation (A9) in Appendix B). According to Simons [48], the relative dielectric permeability in the membrane is essentially smaller than in solution (about 20). Hence, the value of *E_m_* is $\varepsilon /\bar{\varepsilon }$ times smaller than ${\bar{E}}_{m}$.

In the condition of equilibrium between water dissociation and recombination, *R_H/OH_* = 0, and

(7) $$C_{H}C_{OH}=\frac{k_{d}}{k_{r}}C_{w}=K_{w}\approx 10^{-14}{\mathrm{mol}}^{2}L^{-2}.$$

Only the depleted DBL is considered in this mathematical problem. It is a region beyond the water-splitting reaction zone in the membrane, the total flux of water ions ($J_{W}=J_{H}-J_{OH}$) is assumed to be known there. As well, the total current density *I* is given. The partial current densities of the salt counterion (such as the Na^+^ ions) and the product of water splitting (the OH^−^ ions in the case of CEM considered here) are set at the left boundary of the considered region:

(8) $${I_{+}|}_{X=0}=I_{+},\;{I_{OH}|}_{X=0}=I_{w}$$

The quantities *I_+_* and *I_w_* are considered as parameters. Their values can be found experimentally. An example of treatment of the experimental I–V curve, when the partial currents are measured, will be studied below. 

For the sake of simplicity, we assume that the co-ion flux in the membrane and the diffusion layer is zero (*J–* = 0) and the pH value in the solution bulk is 7, the condition for the bulk concentrations being as follows:

(9) $${C_{+}|}_{X=0}={C_{-}|}_{X=0}=C_{1}^{0},\;{C_{H}|}_{X=0}={C_{OH}|}_{X=0}=10^{-7}$$

At the other boundary, *X* = 1, which is the membrane surface, the salt counterion concentration is assumed to be known:

(10) $${C_{+}|}_{X=1}=C_{+m}$$

The problem comprising Equations (2)–(10) is a boundary value problem describing ion transport coupled with the chemical reaction of water splitting. A similar problem was earlier studied numerically by Urtenov et al. [56,68]. The difference in the problem formulation is that in [68], the assumption of zero water splitting expressed by Equation (5) was applied; in [56], the boundary condition for the partial current density of the OH^−^ ions was set at *X* = 1 as and not at *X* = 0, as in this paper. The value of *I_OH_* at *X* = 0 corresponds exactly to the experimentally measured current transferred by the products of water splitting. However, the value of *I_OH_* at *X* = 1 can be different as a number of the H^+^ and OH^−^ ions is generated within the depleted diffusion layer.

The numerical solution of the formulated above boundary-value problem was found using the Comsol Multiphysics software (COMSOL Group, Stockholm, Sweden), version 5.5.

To find an analytical solution, some transformations of the above equations are initially performed to obtain the relations convenient for this purpose.

### 2.2. Transformation and Integration of the Equations. Relationships between the Fluxes

The high concentration of counterions and low concentration of co-ions in the membranes together with the condition of continuity of concentrations (Figure 1b) result in the formation of a thin space charge region at the solution/membrane interface (*X* = δ). The other part of the DBL adjacent to the solution bulk (*X* = 0) remains electroneutral. This peculiarity of the DBL structure is due to the small value of the parameter at the derivative in the Poisson equation (Equation (3) when written in the dimensionless form. The mathematical aspects are discussed in [34,66,68,69,70,71] and others. When the current is zero, the SCR in solution constitutes the interfacial equilibrium DBL. With growing the current, the length of the SCR (*L*) increases. If the current density is less than its limiting value *I_lim_* ($I<I_{lim}=2D_{+}FC_{+}^{0}/\delta$), *L* is of the order of the Debye length:

(11) $$L_{D}=\sqrt{\varepsilon \varepsilon_{0}RT/\left(2C_{+s}z_{+}^{2}F^{2}\right)},$$

determined by the minimum concentration of the counterion in the electroneutral solution near the surface (*C_+s_*) (Figure 1b). If $I\geq I_{lim}$, *L* takes macroscopic values comparable with the DBL thickness *δ* [68,70]. As the analytical solution shows (Appendix D, Equation (A19)), the concentration profile of the salt counterion is linear in the electroneutral zone; its linear extrapolation gives the intersection point with the *X* axis at distance $\delta^{\prime }$ from the bulk solution (X=0).

In the electroneutral part of the diffusion layer, the electroneutrality condition is written as:

(12) $$C_{+}-C_{-}+C_{H}-C_{OH}=0,\;0\leq X\leq \delta^{\prime }.$$

Taking into account that at $X=\delta^{\prime }$ the concentration of all species is small compared to the salt concentration in the bulk, the following inequality may be applied:

(13) $${\left(C_{+}+C_{-}+C_{H}+C_{OH}\right)}_{X=\delta^{\prime }}<<C_{+}^{0}+C_{-}^{0}+C_{H}^{0}+C_{OH}^{0},$$

where $C_{i}^{0}=C_{i}\left(0\right)$.

Dividing each flux in Equation (2) by *D_i_*, including the co-ion flux (*J_–_*) which is assumed zero, and summing the results yields:

(14) $$\frac{J_{+}}{D_{+}}+\frac{J_{H}}{D_{H}}+\frac{J_{OH}}{D_{OH}}=-\frac{d}{dX}\left(C_{+}+C_{-}+C_{H}+C_{OH}\right)+\frac{FE}{RT}\left(C_{+}-C_{-}+C_{H}-C_{OH}\right).$$

By integrating Equation (14) over the electroneutral part of the diffusion layer (where $C_{+}-C_{-}+C_{H}-C_{OH}=0$), one obtains:

(15) $$\frac{J_{+}}{D_{+}}\delta^{\prime }+\int_{0}^{\delta^{\prime }}\left(\frac{J_{H}}{D_{H}}+\frac{J_{OH}}{D_{OH}}\right)dX\approx C_{+}^{0}+C_{-}^{0}+C_{H}^{0}+C_{OH}^{0}.$$

In the considered case of CEM, and OH^−^ ions generated in water splitting at the solution/membrane interface move towards the center of the desalination compartment. When $C_{OH}^{0}\geq C_{H\;}^{0}$ in the bulk, the $C_{OH}>C_{H}$ inequality is valid in the whole electroneutral zone of the DBL. The value of $J_{OH}$ in this region is comparable with *J*_+_, while $J_{H}$ is negligible. Introducing $J_{W}=J_{H}-J_{OH}\approx -J_{OH}$ in Equation (15) leads to Equation (16):

(16) $$J_{+}=\frac{2D_{+}C_{+}^{0}}{\delta^{\prime }}+\frac{D_{+}}{D_{OH}}J_{w}.$$

The difference between Equations (1) and (16) is only in the sense of *δ* and $\delta^{\prime }$: *δ* in Equation (1) is the total thickness of the DBL, $\delta^{\prime }$ is the effective thickness of the DBL, which is approximately equal to the thickness of its electroneutral part (Figure 1b).

### 2.3. Approximate Solution. The Diffusion Layer Structure

#### 2.3.1. Equations in Dimensionless Form 

Equations (2) and (3) in the region 0≤X≤δ near a cation-exchange membrane can be rewritten in the following dimensionless form more convenient for theoretical analysis:

(17) $$j_{k}=-d_{k}\left(\frac{dc_{k}}{dx}-z_{k}c_{k}e\right),\;k=“+”,\;“-”,\;“H”,\;“\mathrm{OH}”;$$

(18) $$\tilde{\varepsilon }\frac{de}{dx}=c_{+}-c—c_{OH,}$$

(19) $$i=j_{+}-j_{-}+j_{H}-j_{OH,}$$

where

(20) $$j_{k}=\frac{J_{k}\delta }{D_{+}C_{+}^{0}},\;i=\frac{I\delta }{FD_{+}C_{+}^{0}},\;c_{k}=\frac{C_{k}}{C_{k}^{0}},\;d_{k}=\frac{D_{i}}{D_{+}},\;x=\frac{X}{\delta }\;,\tilde{\varepsilon }=2{\left(\frac{L_{D}}{\delta }\right)}^{2}.$$

In the considered region, the concentration of H^+^ ions is small as compared to that of the other ions ( $C_{H}\ll C_{k}$) and is neglected as well as the flux of these ions. The flux of salt co-ions is assumed zero (*j_–_* = 0), *j_+_* > 0, and *j*_OH_ < 0 when the current density *I* is positive. The dimensionless values of the limiting salt counterion flux and limiting current density are equal to *j_+lim_* = 2 and *i_lim_* = 2, respectively. $\tilde{\varepsilon }$ is a small parameter, its value ranges from 5 × 10^−12^ to 5 × 10^−5^, the extremum cases being $C_{1}^{0}$ = 1 mol·L^−1^, *δ* = 200 μm, and $C_{1}^{0}$ = 10^−5^ mol·L^−1^, *δ* = 20 μm, respectively.

The problem formulated by Equations (17)–(20) is similar to Rubinstein’s problem [70]; the difference is that we additionally take into account the transport of H^+^ and OH^−^ ions.

#### 2.3.2. Thicknesses of Different Zones. Stitching of Solutions

The problem is solved by the method developed in [64] in the case of a binary electrolyte without water splitting. As earlier [64], it is supposed that the DBL lying between *x* = 0 and *x* = 1 consists of three zones: the electroneutral zone of thickness *δ_1_*; the electromigration zone of space charge region of thickness *δ_2_*, where the diffusion contribution to the fluxes is much smaller than the electromigration contribution; and the quasi-equilibrium part of the SCR adjacent to the membrane surface (quasi-equilibrium electric double layer) of thickness δ*_3_*, the values of fluxes in this zone are much smaller than their diffusion and electromigration components 

Taking into account the peculiarities of each zone described above, it is possible to solve approximately Equations (17)–(19) and express the obtained solutions through one parameter, the minimum salt counterion concentration in the depleted diffusion layer, *c_+s_*. The detailed deduction of the approximate solution of the NPP equations in each zone is presented in the Appendix A, Appendix B, Appendix C and Appendix D. In particular, the following expressions are found for the thicknesses of the mentioned above three zones:

(21) $$\delta_{1}\approx \delta \frac{2}{j}\left(1-c_{+s}\right),$$

(22) $$\delta_{2}\approx \delta \frac{\tilde{\varepsilon }j_{+}^{2}}{2c_{+s}^{2}j},$$

(23) $$\delta_{3}\approx \delta \sqrt{\frac{2\tilde{\varepsilon }}{c_{+s}}},$$

where

(24) $$j=j_{+}+\frac{D_{+}}{D_{OH}}j_{OH},\;j^{\prime }=j_{+}-\frac{D_{+}}{D_{OH}}j_{OH}$$

are the auxiliary parameters, which are calculated through the known values of *j*_+_ and *j_OH_*.

Note that there is a small difference between $\delta_{1}$ and $\delta^{\prime }$ at $i\geq i_{lim}$. $\delta^{\prime }$ is the effective thickness of the diffusion layer. As mentioned above, it is defined as the distance from the solution bulk (*x* = 0) to the point of intersection of the tangent, drawn to the salt counterion concentration profile in the electroneutral zone, with the *c* = 0 axis. It follows from Equation (A19) that $\delta^{\prime }$ = δ⋅2/j. The value of $\delta_{1}$ is determined by the intersection of the same tangent with the straight line *c = c_+s_* (Appendix A). The sense of $\delta_{1}$ and $\delta^{\prime }$ is also clear from Figure 1b. Since at $i\geq i_{lim}$, *c_+s_*<<1, the values of $\delta_{1}$ and $\delta^{\prime }$ are very close.

The stitching of solutions in different zones is made by connecting the expressions for $\delta_{1}$, $\delta_{2}$, and $\delta_{3}$ in one equation, Equation (25), following from the fact that the sum of the thicknesses above is equal to the thickness of the diffusion layer *δ*; the latter is considered as an adjustable parameter:

(25) $$\frac{2}{j}\left(1-c_{+s}\right)+\frac{\tilde{\varepsilon }j_{+}^{2}}{2c_{+s}^{2}j}+\sqrt{\frac{2\tilde{\varepsilon }}{c_{+s}}}=1.$$

Solving Equation (25) for the given values of partial current densities, *j*_+_ and *j_OH_*, yields the value of $c_{+s}$. When knowing $c_{+s}$, it is possible to calculate the potential drops in different zones and in the whole system (see the next section), as well as to find the concentration profiles of all ions present in the system (see Appendix, the last section).

If $i<i_{lim}$, the terms in the left-hand part of Equation (25) containing small parameter $\tilde{\varepsilon }$ can be neglected and Equation (A8) (see Appendix B) is obtained for calculation of $c_{+s}$. If $i\geq i_{lim}$ (*j* > 2), the terms $c_{+s}$ and $\sqrt{2\tilde{\varepsilon }/c_{+s}}$ can be neglected compared to 1, and a simple estimation for the $c_{+s}$ calculation can be used:

(26) $$c_{+s}=\frac{j_{+}\sqrt{\tilde{\varepsilon }/2}}{\sqrt{j-2}}.$$

#### 2.3.3. Potential Drops

Potential drops across different zones of the DBL are found by integrating Equation (A13) (see Appendix C) when making some simplification in each zone. In the electroneutral zone and the electromigration zone of the SCR, the potential drops are, respectively:

(27) $$\Delta \varphi_{1}=\frac{RT}{F}\frac{j^{\prime }}{j}\ln c_{+s},$$

(28) $$\Delta \varphi_{2}=-\frac{RT}{F}\frac{j_{+}{\tilde{\delta }}_{2}}{c_{+s}}\left(1-\frac{j_{+}}{4j}\right).$$

There is no necessity to find the potential drop over the equilibrium part of the EDL, zone of thickness *δ*_3_. Instead, it is possible to find the sum of interfacial potential drops at the left-hand and right-hand membrane boundaries. At the left-hand interface, we consider the region between the point *x* = *x_s_* (Figure 1b) and the nearest point inside the membrane where the electroneutral condition is hold (the left-hand limit of the membrane bulk). The Donnan potential drop there is $\Delta {\varphi^{\prime }}_{D}=-\frac{RT}{z_{+}F}\ln\frac{{{\bar{C}}^{\prime }}_{+}}{C_{+s}}$, where ${\bar{C}}_{+}^{\prime }$ is the concentration of the salt counterion in the membrane bulk next to the left-hand boundary. At the right-hand boundary of the membrane, no space charge appears outside the double electric layer, and the potential drop is expressed by the Donnan equation: $\Delta {\varphi^{\prime \prime }}_{}{}_{D}=-\frac{RT}{z_{+}F}\ln\frac{C_{+s}^{b}}{{{\bar{C}}^{\prime \prime }}_{+}}$, where ${\bar{C}}_{+}^{\prime \prime }$ is the salt counterion concentration in the membrane bulk next to the right-hand boundary, $C_{+s}^{b}$ is the salt counterion boundary concentration in the concentration (brine) compartment. Assuming that ${\bar{C}}_{+}^{\prime }={\bar{C}}_{+}^{\prime \prime }$, the sum of two Donnan potential drops is

(29) $$\Delta \varphi_{D}=\Delta \varphi_{D}^{\prime }+\Delta \varphi_{D}^{\prime }=-\frac{RT}{z_{+}F}\ln\frac{C_{+s}^{b}}{C_{+s}}.$$

The other potential drops (in the membrane bulk, concentrating compartment, etc.) can be easily calculated, if necessary; they are not considered in this paper.

## 3. Experimental CVC Treatment

**Figure 2:**
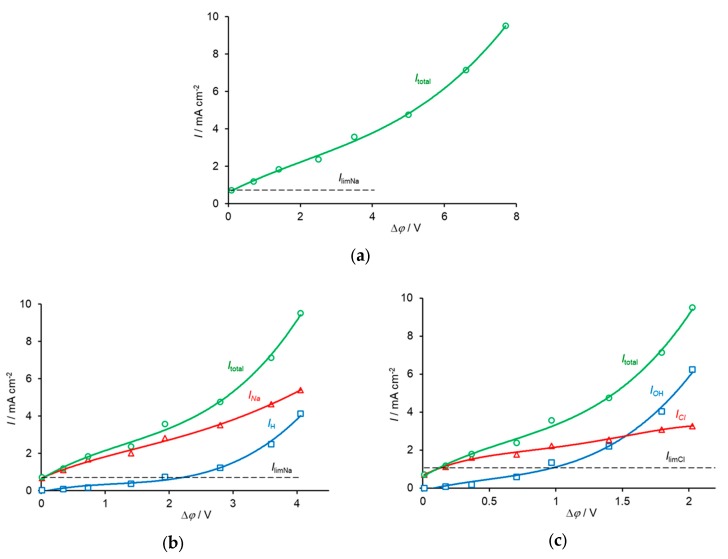
Current–voltage curves for a desalination compartment formed of a MA-40 anion-exchange membrane and a MK-40 cation-exchange membrane. The potential drop across both membranes (**a**) refers to points 1 and 2 in Figure 1a; that across the MK-40 membrane (**b**), to points 3 and 2; that across the MA-40 membrane (**c**), to points 1 and 3. In the case of individual membranes, the potential drop in the bulk solution was subtracted. The total and partial currents of the Cl^−^ and OH^−^ ions through the MA-40 membrane and Na^+^ and H^+^ ions through the MK-40 membrane are shown. The symbols show the experimental data taken from [72]; the lines are a guide to the eye.

Figure 2 shows the current–voltage curves (CVC) measured for MA-40 anion-exchange and MK-40 cation-exchange membranes both produced by Shchekinoazot, Russia. 

The membranes form a cell-pair in an ED cell, schematically shown in Figure 1a. The active membrane area was 3 cm × 3 cm, the distance between the membranes was 0.1 cm. A 0.002 M NaCl solution with an average velocity of 3.2 cm·s^−1^ passed between the membranes. The potential drop across the cell-pair was measured using two Luggin capillaries whose positions are shown with points 1 and 2 in Figure 1b. The total and partial current densities of the Na^+^ and H^+^ ions through the MK-40 membrane and those of the Cl^−^ and OH^−^ ions through the MA-40 membrane were found [72] by using the constant pH method, where the pH of the feed solution circulating through the desalination compartment was maintained constant [73]. The partial fluxes/currents were calculated, knowing the rate of decrease in the conductivity of the diluate over time and the rate of addition of a NaOH solution to the diluate, which was necessary to maintain its pH constant.

A very small distance between the membranes did not allow to put a capillary in the desalination compartment. However, it was of interest to evaluate the potential drops across the AEM (between points 1 and 3 in Figure 1a) and CEM (between points 3 and 2). Since the mobility of Cl^−^ ion, which was the counterion for the AEM, was approximately 1.5 times higher than that of Na^+^, the counterion for the CEM, one can expect that at the same current density, the potential drop across the AEM will be lower than across the CEM. Following [68], we assumed that 2/3 of the total potential drop referred to the CEM and 1/3, to the AEM. To meet the conditions of the model, Equations (27)–(29), the potential drop in the bulk solution of the desalination compartment was subtracted.

The procedure of the data treatment at any overlimiting current density is the same when applying the analytical or numerical solution. Knowing the partial current densities of the salt counterion (*J*_+_ in the case of CEM, *J*_−_ in the case of AEM) and the H^+^ and OH^−^ ions in the CEM and AEM, respectively, it is possible to calculate the effective thickness of the diffusion layer, $\delta^{\prime }$, by using Equation (1) for each membrane. Then the value of the diffusion layer thicknesses, *δ*, for the AEM and CEM, are found by fitting the experimental values of the potential drop across the AEM and CEM, respectively.

**Figure 3:**
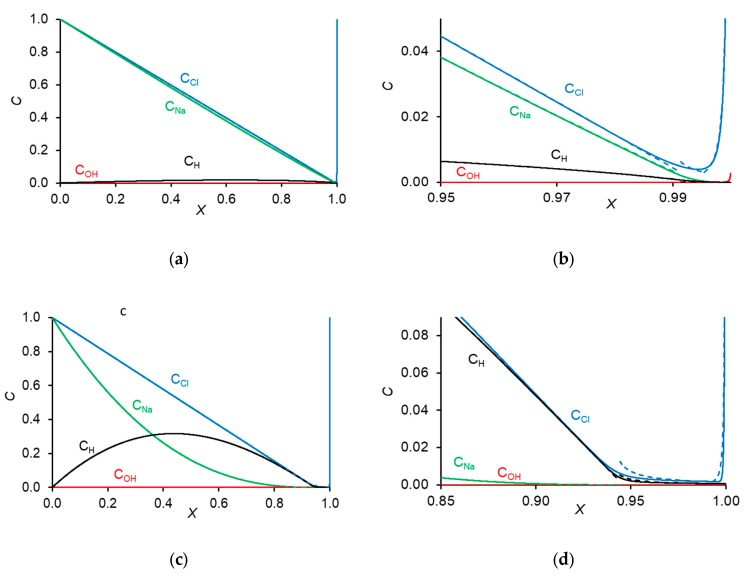

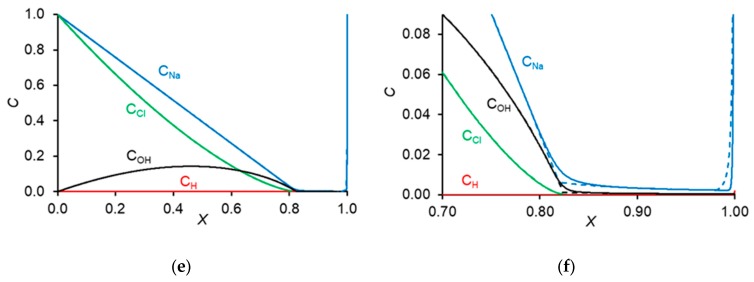
Concentration profiles of Na^+^, Cl^−^, H^+^, and OH^−^ ions in the depleted DBL near the AEM (**a**–**d**) and CEM (**e**,**f**) shown at different scales, at current densities *I* = 1.8 mA cm^−2^ (**a**,**b**) and *I* = 9.5 mA cm^−2^ (**c**–**f**). Solid and dashed lines were calculated numerically and analytically, respectively. *X* = 0 relates to the bulk solution, *x* = 1, to the membrane surface.

Figure 3a–f shows the concentration profiles calculated numerically and analytically in the depleted DBL near an AEM and CEM, respectively. 

In calculations, the following parameters were used: the diffusion coefficients for Na^+^, Cl^−^, H^+^, and OH^−^ ions were taken as 1.33 × 10^−5^, 2.05 × 10^−5^, 9.31 × 10^−5^, and 5.27 × 10^−5^ (in cm^2^·s^−1^); bulk solution conductivity κ_sol_ = 0.025 S·m^−1^; the ohmic potential drop in the membranes was neglected, taking into account their high conductivity, about 0.4 S·m^−1^, compared to that of the solution. The limiting current density through the AEM and CEM was determined from the experimental CVC (by the point of intersection of the tangents drawn to the initial region and to the inclined plateau of the CVC); it was equal approximately to 0.72 mA·cm^−2^ and 1.1 mA·cm^−2^, respectively. According to the Peers equation, which is a particular case of Equation (1) when *I_w_* = 0, these values of the limiting current density refer to a DBL with a thickness of 72 µm.

**Figure 4:**
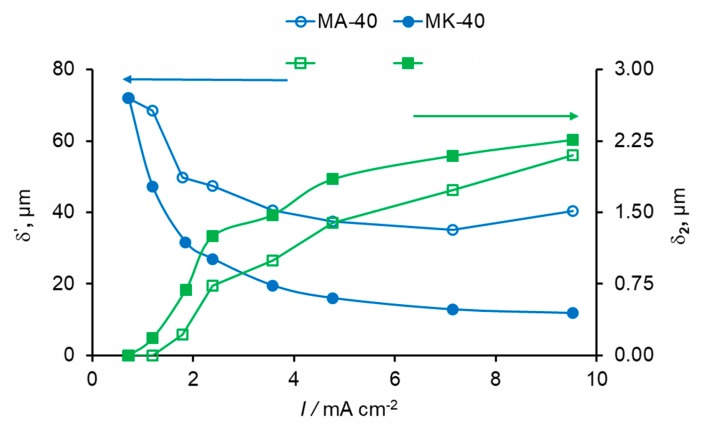
The effective thicknesses of the diffusion layer, δ′, and the thickness of the extended SCR, *δ*_2_, as functions of the applied current density, for the AEM and CEM.

First of all, we note a very good agreement between the analytical and numerical solutions. Within each zone, perhaps with the exception of the quasi-equilibrium part of the DBL, the curves calculated analytically follow well the numerically computed curves. However, in the places of joining two zones, the analytical curve, describing the concentration profile within one zone, continues its trend outside of this zone, while the numerically found curve changed its course. It can also be seen that at the same current density, the thickness of the extended SCR was significantly larger near the CEM than AEM; see also Figure 4. 

**Figure 5:**
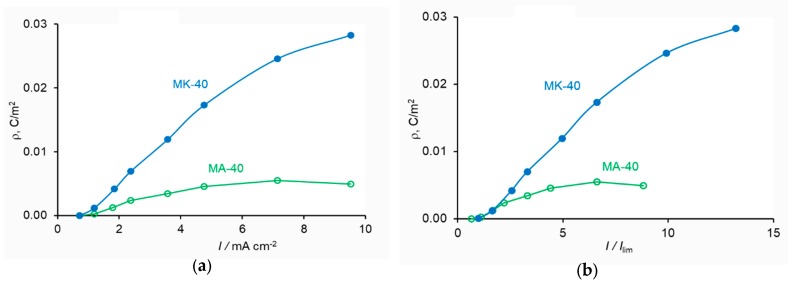
The extended space charge surface density as a function of the applied current density (**a**) and the *I*/*I*_lim_ ratio (**b**), for the AEM and CEM. Numerical calculation.

The effective thickness of the diffusion layer, δ′, near the CEM was essentially lower than that near the AEM at a given current density. This shows that electroconvective mixing of depleted solution, which is the cause of the decrease of δ′, should be more intensive near the CEM. This correlates with the fact that the thickness of the extended SCR, *δ*_2_, was larger near the CEM. Moreover, the space charge surface density localized in the depleted solution near the CEM was significantly lower than that near the AEM (Figure 5).

This model allows obtaining a better understanding of the questions frequently discussed in the literature [30,53,54,55,74] concerning the role of water splitting in the salt ion transport through ion-exchange membranes. Although it is well established that the reason for the higher WS rate at an AEM is the presence of functional groups (most often tertiary and secondary amino groups), which catalytically affect WS through proton transfer reactions [28,48], the causes of less intensive EC at an AEM compared with CEM are poorly understood.

As Equation (1) shows, there are formally two ways for enhancement of the salt counter-ion transfer: either to decrease the effective thickness of the DBL ($\delta^{\prime }$), or to increase the water splitting rate (*I_w_*). Increasing *I_w_* leads to a higher exaltation effect, when the salt counterions are attracted from the solution bulk to the membrane surface by the H^+^/OH^−^ ions generated in WS. One of the possible mechanisms for decreasing $\delta^{\prime }$ is electroconvection, which is the most effective in diluted solutions where the EDL thickness is relatively large [75]. The electric current acts on the space charge in the depleted solution near the interface and makes the SCR in motion producing EC, which destroys the DBL from the inside [22,38,40,41]. The more the space charge and the SCR thickness, the more the effect of this motion on the transport enhancement and less $\delta^{\prime }$. The space charge and the SCR thickness increase with increasing voltage, as a growing electric field more effectively removes the co-ions from the depleted solution and draws the counterions into this near-surface region. 

As for the exaltation effect, its influence on the increasing salt counterion transfer is rather low [29], as the ratio *D_+(−)_/D_w_* is of the order of 10^−1^. For example, when *I_w_* = *I*_+(−)_ (hence, 50% of the charge is transferred by the H^+^ and OH^−^ ions), the increase in *I*_+(−)_ due to the exaltation effect is only about 10%, according to Equation (1). Moreover, as it was found in the literature [29,30], and shown by the experimental data presented in this paper, WS essentially reduces EC.

The treatment of experimental partial current–voltage curves using the developed model shows that under the same current density, the surface space charge density in the depleted DBL at the AEM is lower than that at the CEM. This should be due to the fact that the WS rate at the AEM is significantly higher than that at the CEM. A higher WS rate results in a higher concentration of the H^+^ ions in the extended SCR near the AEM in comparison to the concentration of the OH^−^ ions in the SCR near the CEM (compare Figure 3d,f). Since the charge of the H^+^ ions is opposite to that of the space charge at the AEM (which is determined by the salt anions), the presence of the H^+^ ions reduces the space charge density. The lower space charge density results in a less intensive EC. Note that this explanation was previously proposed by Mishchuk based on a theoretical consideration [74]. The use of a mathematical model applied to quantitative processing of experimental data provides more arguments to this explanation.

The increment of the current density transferred by the products of water splitting within the solution DBL, *I*_w_^s^, can be estimated according to [48] and Equation (5) as

(30) $$I_{w}^{s}=Fk_{d}C_{w}\delta_{SCR},$$

where *k*_d_ is the water dissociation constant in the solution SCR, *C*_w_ is the water concentration, *δ*_SCR_ = *δ*_2_ + *δ*_3_ is the SCR thickness.

**Figure 6:**
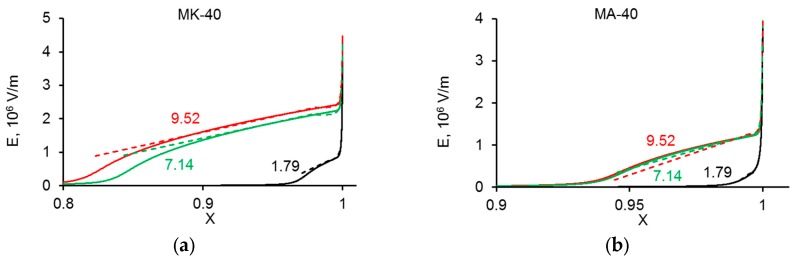
The electric field in the SCR of the depleted solution at the CEM (**a**) and AEM (**b**) as a function of the distance at different current densities, the values of which in mA·cm^−2^ are shown near the curves; *x* = 0 relates to the bulk solution, *x* = 1, to the membrane surface. The solid lines are calculated numerically, the dashed lines, using the analytical solution, Equation (A31).

**Figure 7:**
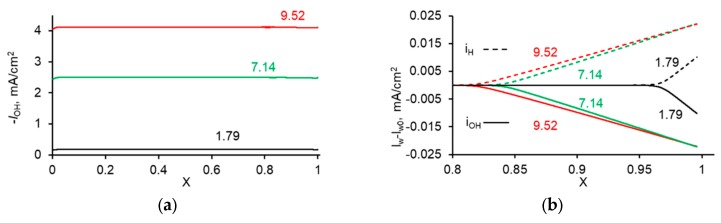
The partial current density of the OH^−^ ions (**a**) and the increment, *I_w_*−*I_w0_*, of the current densities of the H^+^ and OH^−^ ions, *I_w_*, over their values at *x* = 0, *I_w0_*, as functions of the distance, *x*, in the depleted diffusion layer at the CEM (**b**) at different current densities; *x* = 0 relates to the bulk solution, *x* = 1, to the membrane surface. The values of the current density in mA cm^−2^ are shown near the curves.

**Figure 8:**
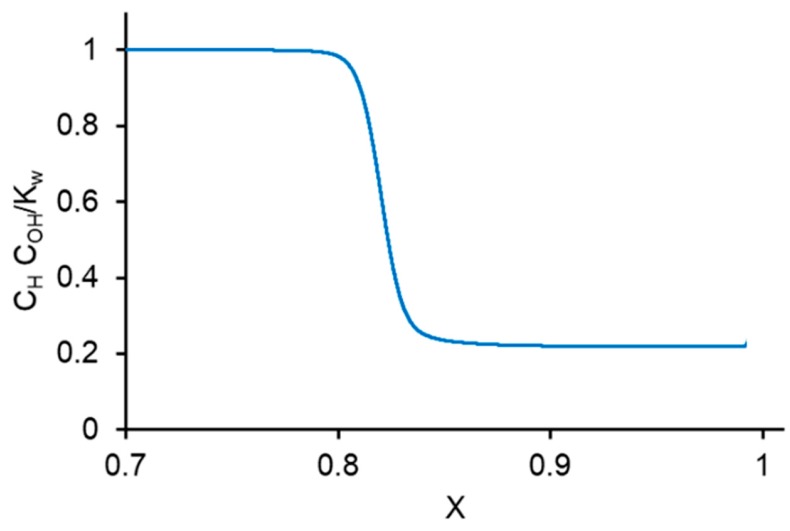
The *C_H_C_OH_/K_w_* ratio as a function of coordinate *x* for the case of CEM and *I* = 9.5 mA·cm^−2^. Numerical computation.

This increment is due to the formation of the H^+^ and OH^−^ ions in the depleted diffusion layer by water dissociation. The electric field in this SCR is not so strong (of the order of 10^6^ V·m^−1^, Figure 6), as in the interface layer of a bipolar membrane, where it is of the order of 10^8^ V m^−1^ [76]. For this reason, the Wien effect described by Equation (6) is not significant in the depleted diffusion layer at a monopolar membrane. Hence, it is possible to set *k*_d_ = 2 × 10^−5^ s^−1^ in the SCR. The comparison of the calculations using Equation (30) with the numerical solution shows that Equation (30) gives a slightly overestimated value for *I*_w_^s^. For example, at *I* = 9.5 mA·cm^−2^, the *δ*_SCR_ value was 2.26 µm. Setting *C*_w_ = 55.6 mol·L^−1^, we found the increase of *I_w_* within the depleted solution *I*_w_^s^ ≈ 0.030 mA·cm^−2^. The numerical solution gives 0.021 mA·cm^−2^ (Figure 7b). The difference is due to the fact that the product *C*_H_*C*_OH_ in the SCR is not much less than *K*_w_ (as it is assumed when obtaining Equation (30)), but is comparable with *K*_w_ (Figure 8). The value of *I*_w_^s^ is much lower than the experimental value of *I_w_* = 4.1 mA·cm^−2^. This means that nearly all the H^+^ and OH^−^ ions were generated in the interfacial layer of the membrane, where the increase in *I_w_* was equal to 4.05 mA·cm^−2^ (Figure 7a).

To understand whether the WS in the depleted diffusion layer is important or not, it is also necessary to compare the value of *I*_w_^s^ with the current density transferred by salt counterions. In the considered case, the solution (a 0.002 M NaCl) if rather dilute, but the salt counterion current density, *I_+_*, was relatively high, 5.4 mA cm^−2^. It is due to good hydrodynamic conditions in the experimental cell and intensive EC, which provide a very thin diffusion layer, of the thickness of about 14 µm. However, if the conditions for salt ion transfer are not so favorable, and the feed solution concentration is lower, the WS in the depleted solution may be significant. For example, in the case of a 5 × 10^−4^ M NaCl and a diffusion layer thickness *δ* = 100 µm, the limiting current density *I*_lim_ found as 2 *D*_+_*F*$C_{+}^{0}$/*δ* (the Peers equation) was approximately equal to 0.13 mA·cm^−2^. Then *I*_w_^s^ = 0.02 mA·cm^−2^ becomes a significant contribution to the total current, which can noticeably change the pH of the diluate/concentrate solutions.

According to the developed model, the greater the parameter $\tilde{\varepsilon }$, the larger the relative thickness of SCR, hence the stronger can be expected electroconvection. As Equation (20) shows, $\tilde{\varepsilon }$ increases with increasing *L_D_* and decreasing *δ*. *L_D_* increases with decreasing the solution concentration; hence, it can be predicted that electroconvection enhances with diluting the feed solution. The situation is more complicated concerning the value of *δ*. This value can be decreased when increasing the mean velocity of the feed solution forced flow in the desalination compartment. However, a greater forced flow rate could hinder the development of electroconvection. As for the rate of water splitting, it should increase when the feed solution concentration decreases, and decrease with increasing electroconvection. The latter was indirectly shown experimentally [46]. Note that neither the effect of concentration nor the influence of flow velocity has been studied systematically in the literature. Nevertheless, this topic seems important and interesting both for the theory and practice of electrodialysis. 

## 4. Conclusions

The semi-analytical mathematical description of transfer of salt and H^+^ (OH^−^) ions in the depleted diffusion layer near an ion-exchange membrane offers a relatively easy way to obtain important information about the distribution of ions and electric field in the vicinity of the membrane. The knowledge of the local values of ion concentrations (e.g., of the OH^−^ and doubly charges cations) allows one to predict whether the precipitation of the sparingly soluble compounds will occur. The model also makes it possible to calculate the potential drop across an ion-exchange membrane on the conditions that the partial current densities of the salt and water ions are specified. The treatment of experimental partial current–voltage curves shows that under the same current density, the surface space charge density in the solution at the anion-exchange membrane was lower than that at the cation-exchange membrane. This should be due to the fact that the WS rate at the AEM was significantly higher than that at the CEM. A higher WS rate results in a higher concentration of the H^+^ ions, which are co-ions, in the extended SCR at the AEM. This reduces the space charge density and partially suppresses EC. Thus, water splitting not only reduces the current efficiency and increases the risk of precipitation, but also reduces the mass transfer through suppressing electroconvection.

The water splitting occurring in the depleted diffusion layer, where there is no catalytic effect, is insignificant in most cases. This process should be taken into account only in quite dilute solutions and in the presence of a thick diffusion layer, when the salt ion transfer through the membrane is small. The analytical evaluation of the water-splitting rate in the depleted solution is overestimated since the *C_H_C_OH_* product remains comparable with the *K_w_* value in the solution space charge region where water splitting takes place.

## Appendix A. Thickness of Different Zones of the Depleted Diffusion Layer

Equation (14) in the region [0, 1] rewritten in dimensionless variables in accordance with Equation (20), has the following form:

(A1) $$j=-d\left(c_{+}+c_{-}+c_{OH}\right)/dx+e\left(c_{+}-c_{-}-c_{OH}\right),$$

where the concentration and flux of H^+^ ions are neglected. By integrating Equation (A1) from *x* = 0 to *x = x_s_* (*x_s_* is the point where $dc_{+}/dx=0$, Figure 1b) while expressing ($c_{+}-c_{-}-c_{OH}$) from Equation (18) and presenting *ede/dx* as (½)*de*^2^/*dx*, we obtain:

(A2) $$jx_{s}=\left(c_{+}^{0}+c_{-}^{0}+c_{OH}^{0}\right)-\left(c_{+s}+c_{-s}+c_{OHs}\right)+\frac{\tilde{\varepsilon }}{2}\left(e_{s}^{2}-e_{0}^{2}\right),$$

where subscript “*s*” denotes the value related to point *x_s_*, index “0” corresponds to *x* = 0. At point *x_s_* as follows from Equation (17) and condition $dc_{+}/dx=0$, the electric field is

(A3) $$e_{s}=j_{+}/c_{+s},.$$

Assuming that at *x* = 0 the concentrations $c_{H}^{0}=$$c_{OH}^{0}\ll$$c_{+}^{0}$,$c_{-}^{0}$, we find in dimensionless variables:

(A4) $$c_{+}^{0}+c_{-}^{0}+c_{OH}^{0}\;\approx \;2.$$

At an underlimiting current (*j* < 2), the electric field $e_{s}$ is not too high because $c_{+s}$ is not very small, and the term in the right-hand part of Equation (A2) containing small parameter $\tilde{\varepsilon }$ may be neglected. In this case, the thickness of the SCR is small compared to the diffusion layer thickness and $x_{s}\approx 1$. When taking into account the electroneutrality condition and the fact that water splitting is neglectable at *j* < 2, we can assume $c_{OHs}\ll c_{+s}\approx c_{-s}$. Then it follows from Equation (A2) that the salt counterion concentration at point *x_s_* is

(A5) $$c_{+s}=1-\left(\frac{j}{2}\right).$$

At overlimiting currents (*j* ≥ 2), the sum of concentrations in Equation (A2) at *x = x_s_* is small compared to that in the bulk (*x* = 0); however, the term $\tilde{\varepsilon }\left(e_{s}^{2}-e_{0}^{2}\right)/2$ in this equation cannot be neglected. Nevertheless, electric field $e_{s}$ at *x = x_s_* is great compared to the $e_{0}$ value, which allows obtaining the following expression from Equation (A2):

(A6) $$x_{s}=\frac{2}{j}+\frac{\tilde{\varepsilon }}{2}\frac{j_{+}^{2}}{jc_{+s}^{2}}.$$

For calculation of $c_{+s}$ at any current, Equation (A6) can be presented as following approximated equation:

(A7) $$\frac{2}{j}\left(1-c_{+s}\right)+\frac{\tilde{\varepsilon }}{2}\frac{j_{+}^{2}}{c_{+s}^{2}j}=x_{s},$$

which is transformed into Equation (A5) at *j* < 2 and into Equation (A6) at j≥2.

Equation (A7) can be interpreted as equality of the sum of the dimensionless thickness of electroneutral zone (${\tilde{\delta }}_{1}$) and that of the space charge region (${\tilde{\delta }}_{2}$) to *x_s_*. The expressions for ${\tilde{\delta }}_{1}$ and ${\tilde{\delta }}_{2}$ in dimension form are presented by Equations (21) and (22), respectively.

Equation (A7) may be completed by adding the thickness of the quasi-equilibrium double electric layer ${\tilde{\delta }}_{3}\approx \sqrt{2\tilde{\varepsilon }/c_{+s}}$ (see the derivation below in Appendix D. Concentration profiles). The sum of the thicknesses of all three zones of the diffusion layer is equal to 1 in dimensionless variables, then Equation (25) is obtained. Note that usually ${\tilde{\delta }}_{3}$ is small compared to the DBL thickness and may be neglected except in the case of very diluted solutions.

## Appendix B. Space Charge and Relation between the Strengths of Electric Field in the Membrane and Solution

The right-hand side of Equation (3) represents the space charge density (ρ). Its integration from *X* = 0 to *X* = *δ* gives the dimensionless space charge localized in the depleted diffusion layer:

(A8) $$q=\int_{0}^{\delta }\rho dx=\int_{0}^{\delta }\varepsilon \varepsilon_{0}\frac{dE}{dX}dX=\varepsilon \varepsilon_{0}\;\left[E\left(\delta \right)-E\left(0\right)\right]\varepsilon \;\varepsilon_{0}E_{m},$$

where the electric field in the bulk $E_{0}$ is neglected compared to *E_m_* = *E*(*δ*).

On the other hand, a similar expression can be obtained for the space charge $\bar{q}=\varepsilon \;\varepsilon_{0}{\bar{E}}_{m}$ located in the boundary layer of the membrane. Since, the global electroneutrality holds, we find that

(A9) $$\varepsilon E_{m}=\bar{\varepsilon }{\bar{E}}_{m}.$$

## Appendix C. Potential Drops in Different Zones

The integration of Equation (A1) from *x* = 0 to arbitrary *x* similarly as in Appendix A gives:

(A10) $$c_{+}+c_{-}+c_{OH}=\left(2-jx\right)+\frac{\tilde{\varepsilon }}{2}\left(e^{2}-e_{0}^{2}\right).$$

After multiplying Equation (A10) by *e*, we find:

(A11) $$\left(c_{+}+c_{-}+c_{OH}\right)e=\frac{\tilde{\varepsilon }}{2}e^{3}-\left(\frac{\tilde{\varepsilon }}{2}e_{0}^{2}+jx-2\right)e.$$

On the other hand, dividing each flux in Equation (17) by *d_k_* and finding $\frac{j_{+}}{d_{+}}-\frac{j_{-}}{d_{-}}-\frac{j_{OH}}{d_{OH}}$ yield:

(A12) $$\left(c_{+}+c_{-}+c_{OH}\right)e=\left(j_{+}-\frac{D_{+}}{D_{OH}}j_{OH}\right)+\frac{d\left(c_{+}-c_{-}-c_{OH}\right)}{dx}=j^{\prime }+\tilde{\varepsilon }\frac{d^{2}e}{dx^{2}}.$$

By comparing Equations (A11) and (A12), and neglecting small quantity $\frac{\tilde{\varepsilon }}{2}e_{0}^{2}$, we obtain:

(A13) $$\tilde{\varepsilon }\frac{d^{2}e}{dx^{2}}=\frac{\tilde{\varepsilon }}{2}e^{3}-\left(jx-2\right)e-j^{\prime }.$$

The integration of Equation (A13), in general case, permits finding the electric field as a function of *x* and then the potential drops in different zones of the depleted diffusion layer. However, the integration of this equation in different zones of the diffusion layer gives fairly simple approximate expressions, when the features of each zone are taken into account.

### Appendix C.1. Electroneutral Zone ($0\leq x\leq {\tilde{\delta }}_{1}$)

In this zone $\tilde{\varepsilon }\frac{d^{2}e}{dx^{2}}$ and $\frac{\tilde{\varepsilon }}{2}e^{3}$ are small, hence

(A14) $$e=\frac{j^{\prime }}{2-jx}.$$

The dimensionless potential drop (normalized by *RT/F*) in this zone is

(A15) $$\Delta \Psi_{1}=-\int_{0}^{{\tilde{\delta }}_{1}}edx=\frac{j^{\prime }}{j}\ln\frac{2-j{\tilde{\delta }}_{1}}{2}=\frac{j^{\prime }}{j}\ln c_{+s},$$

when applying Equation (21) for $\delta_{1}$.

### Appendix C.2. Electromigration Space Charge Region (${\tilde{\delta }}_{1}\leq x\leq x_{s}$)

In this zone, the concentrations vary slowly, the quantity $\tilde{\rho }\left(x\right)=c_{+}\left(x\right)-c_{-}\left(x\right)-c_{OH}\left(x\right)$ varies even less and can be approximated as constant approximately equal to $c_{+s}$. The latter approximation constitutes the condition of quasi-uniform distribution of the space charge density (QCD), which can replace the electroneutrality condition in overlimiting current regimes [68]. When applying the QCD condition, we find *e* as a linear function of *x* within zone ${\tilde{\delta }}_{1}\leq x\leq x_{s}$:

(A16) $$e\left(x\right)=e_{s}-\frac{c_{+s}}{\tilde{\varepsilon }}\left(x_{s}-x\right),$$

where for the sake of simplicity, only the charge of the salt counterion is taken into account, the contribution of the water ions being small regarding their high mobility and lower fluxes. Hence, the dimensionless potential drop in this zone is

(A17) $$\Delta \Psi_{2}=-{\tilde{\delta }}_{2}\left(e_{s}+e_{\delta_{1}}\right)/2.$$

By substituting $e_{s}=\frac{j_{+}}{c_{+s}}$ (from Equation (A3)), $e_{\delta_{1}}=\frac{j_{+}}{c_{+s}}\left(1-\frac{j_{+}}{2j}\right)$ (from Equation (A16)), we find

(A18) $$\Delta \Psi_{2}=-\frac{j_{+}{\tilde{\delta }}_{2}}{c_{+s}}\left(1-\frac{j_{+}}{4j}\right).$$

The derivation for the potential drops at the membrane boundaries is presented in the main text.

## Appendix D. Concentration Profiles

### Appendix D.1. Electroneutral Zone ($0\leq x\leq {\tilde{\delta }}_{1}$)

The hydrogen concentration in this zone is negligible: *c_H_* << 1. The profile of counter-ion concentration is obtained from Equation (A2) by applying the electroneutrality condition ($c_{+}=c_{-}+c_{OH}$):

(A19) $$c_{+}\left(x\right)=1-\frac{j}{2}x.$$

For the co-ion, it follows from the Nernst–Planck equation that

(A20) $$\ln c_{-}\left(x\right)=-\int_{0}^{x}e\left(x\right)dx.$$

When taking into account Equations (A14), (A19), and (A20), we find

(A21) $$c_{-}\left(x\right)={\left[c_{+}\left(x\right)\right]}^{j^{\prime }/j}.$$

The concentration of the hydroxyl is calculated from the electroneutrality condition:

(A22) $$c_{OH}=c_{+}-c_{-}.$$

### Appendix D.2. Electromigration Space Charge Region (${\tilde{\delta }}_{1}\leq x\leq x_{s}$)

In this region, the diffusion contribution to ion transport is negligible. Neglecting the diffusion term compared to the migration one in the Nernst–Planck equation (Equation (17)) leads to the following approximations

(A23) $$c_{+}\left(x\right)\approx \frac{j_{+}}{e}\approx \frac{j_{+}}{e_{s}-c_{+s}\left(x_{s}-x\right)/\tilde{\varepsilon }}\;,\;c_{OH}\left(x\right)\approx \frac{-j_{OH}}{e}\approx \frac{-j_{OH}}{e_{s}-c_{+s}\left(x_{s}-x\right)/\tilde{\varepsilon }},$$

where the electric field, *e(x)*, is calculated using Equation (A16).

There is a relationship between *c*_+_ and *c*_OH_ in this region, following from neglecting the diffusion terms in the Nernst–Planck equation:

(A24) $$\frac{c_{OH}}{c_{+}}\approx \frac{D_{+}T_{OH}}{D_{OH}T_{+}},$$

where $T_{+}$ and $T_{OH}$ are the effective transport numbers of the salt counterion and the OH^−^ ions in the depleted diffusion layer, respectively. These transport numbers are determined from the experimental partial current densities: $T_{+}=I_{+}/I$, $T_{OH}=I_{OH}/I$. Note that the transport number of H^+^ ions in the CEM is equal to the transport number of OH^−^ ions in the depleted solution.

The concentrations of salt co-ions and H^+^ ions in this region are negligible.

### Appendix D.3. Equilibrium Electric Double Layer ($x_{s}\leq x\leq 1$)

In this layer, the value of the salt counterion flux density, *j_+_*, in Equation (17), is much smaller than two of its contributions, the diffusion and migration ones. Then we obtain:

(A25) $$\frac{dc_{+}}{dx}=c_{+}e.$$

In the considered layer, only the concentration of the salt counterion (cation in the case of CEM) is significant: the salt anions and OH^−^ are co-ions, their concentration is very low, and the concentration of the H^+^ ions is low because they are generated in the membrane. In these conditions, the Poisson equation (Equation (18)) can be written as

(A26) $$\varepsilon \frac{de}{dx}=c_{+}.$$

Differentiating Equation (A26) and substituting the result in Equation A25 instead of $\frac{dc_{+}}{dx}$, and putting in this equation $\varepsilon \frac{de}{dx}$ instead of *c*_+_, we find:

(A27) $$\frac{de}{dx}e=\frac{d^{2}e}{dx^{2}}.$$

The first and the second integrations of Equation (A27) give, respectively

(A28) $$\frac{de}{dx}=\frac{e^{2}}{2},$$

(A29) $$e\left(x\right)={\left[\frac{1}{e_{m}}+\frac{1}{2}\left(1-x\right)\right]}^{-1},$$

where *e_m_* = *e*(1) is the value of the electric field at the membrane surface.

When expressing *c*_+_(*x*) through the derivative of the electric field, Equation (A26), which is found using Equation (A29), we find an equation for the calculation of $c_{+}\left(x\right)$ in the range $x_{s}\leq x\leq 1$:

(A30) $$c_{+}\left(x\right)=\frac{\varepsilon }{2}{\left[\frac{1}{e_{m}}+\frac{1}{2}\left(1-x\right)\right]}^{-2}.$$

The value of *e_m_* can be evaluated using the first integral of the Nernst–Planck–Poisson equations for a binary electrolyte [64,77], which in the used designations can be written as:

(A31) $$c_{+}+c_{-}=\frac{\varepsilon }{2}e^{2}-\left(jx-2\right).$$

At the membrane surface, *x* = 1, *c*_−_ << *c*_+_=*c*_+m_. As the dimensionless limiting current density, *I*, is close to its limiting value 2, the (*I*−2) term is small, and we find

(A32) $$e\left(1\right)=e_{m}\approx \sqrt{\frac{2c_{+m}}{\varepsilon }},$$

When applying Equation (A30) at point *x* = *x_s_*, where *c*_+_ = *c*_+s_, it is possible to express the thickness of the equilibrium part of the EDL, *δ*_3_, as

(A33) $$\delta_{3}\approx \delta \left(\sqrt{\frac{2\tilde{\varepsilon }}{c_{+s}}}-\sqrt{\frac{2\tilde{\varepsilon }}{c_{+m}}}\right).$$

Since *c*_+m_ >> *c*_+s_, the second term in the right-hand side of Equation (A33) can be neglected, and we arrive at Equation (23).
